# The N-terminal domain plays a crucial role in the structure of a full-length human mitochondrial Lon protease

**DOI:** 10.1038/srep33631

**Published:** 2016-09-16

**Authors:** Sami Kereïche, Lubomír Kováčik, Jan Bednár, Vladimír Pevala, Nina Kunová, Gabriela Ondrovičová, Jacob Bauer, Ľuboš Ambro, Jana Bellová, Eva Kutejová, Ivan Raška

**Affiliations:** 1 Institute of Cellular Biology and Pathology, First Faculty of Medicine, Charles University in Prague, Albertov 4, 128 01 Prague 2, Czech Republic; 2 Université de Grenoble Alpes,CNRS UMR 5309, 38042 Grenoble Cedex 9, France; 3 Department of Biochemistry and Structural Biology, Institute of Molecular Biology, Slovak Academy of Sciences, Bratislava, Slovakia; 4 Institute of Microbiology, Academy of Sciences of the Czech Republic, Prague, Czech Republic; 5 Biomedicine Center of the Academy of Sciences and Charles University in Vestec, Czech Republic.

## Abstract

Lon is an essential, multitasking AAA^+^ protease regulating many cellular processes in species across all kingdoms of life. Altered expression levels of the human mitochondrial Lon protease (*h*Lon) are linked to serious diseases including myopathies, paraplegia, and cancer. Here, we present the first 3D structure of full-length *h*Lon using cryo-electron microscopy. *h*Lon has a unique three-dimensional structure, in which the proteolytic and ATP-binding domains (AP-domain) form a hexameric chamber, while the N-terminal domain is arranged as a trimer of dimers. These two domains are linked by a narrow trimeric channel composed likely of coiled-coil helices. In the presence of AMP-PNP, the AP-domain has a closed-ring conformation and its N-terminal entry gate appears closed, but in ADP binding, it switches to a lock-washer conformation and its N-terminal gate opens, which is accompanied by a rearrangement of the N-terminal domain. We have also found that both the enzymatic activities and the 3D structure of a *h*Lon mutant lacking the first 156 amino acids are severely disturbed, showing that *h*Lon’s N-terminal domains are crucial for the overall structure of the *h*Lon, maintaining a conformation allowing its proper functioning.

Human Lon (*h*Lon, P36776) is a mitochondrial AAA^+^ protein (ATPases Associated with diverse cellular Activities) belonging to the LonA protease subfamily^1^, which plays a crucial role in the maintenance of mitochondrial homeostasis. Its primary function is the degradation of misfolded, oxidatively modified and regulatory proteins^2^, but it also participates in the maintenance of mitochondrial DNA^3^ and possesses a chaperone activity important for the proper assembly of protein complexes^4^. Changes in *h*Lon expression have been linked to severe diseases, including epilepsy, myopathy, paraplegia, and cancer^5^. In several cancerous tissues, overexpression of *h*Lon promoted proliferation of cancer cells^6^ by remodeling their mitochondrial functions^7^ while its down-regulation led to apoptosis and cell death^8^. Silencing of *h*Lon or pharmacologically inhibiting its activity has therefore been considered as a new target for the development of anticancer drugs^9^.

Like other ATPases, Lon’s activities are accompanied by conformational changes induced by ATP binding and hydrolysis^10^,^11^. Early biochemical studies revealed that the binding of protein substrates by Lon stimulates its ATPase and peptidase activities and that this activation is likely to be allosteric^12^,^13^. Menon and Goldberg^12^ first suggested a substrate-induced proteolytic mechanism, in which the default state of Lon is its inactive, ADP-bound form preventing accidental degradation of cellular proteins. Upon substrate binding, this form releases its ADP molecules and binds ATP, which is followed by its rapid hydrolysis and the cleavage of peptide bonds. In this mechanism, Lon can bind and hydrolyze ATP as long as the substrate binding sites are occupied. More recently, the idea that Lon’s ATPase and protease activities are under allosteric control has been supported by degron binding studies^14^,^15^, and Su *et al*.^16^ have very recently found that binding of Mg^2+^ ions to Lon protease from *M. taiwanensis (Mta*Lon) induces conformational changes in Lon’s AP-domain that accompany ATP-independent partial proteolysis of unfolded proteins and cleavage of specific peptides.

Therefore, the Lon protease is a dynamic protein whose functional domains (proteolytic, ATPase-, and N-terminal domains) are present on a single polypeptide chain^1^. This arrangement distinguishes it from most other AAA^+^ proteases; the exceptions are the bacterial FtsH and *m*-AAA proteases^17^,^18^,^19^. To date, a full-length three-dimensional Lon structure has not been determined, and therefore its complex mechanisms of action are only partially understood. Although several LonA X-ray crystal structures have been reported to date, some of them representing a substantial portion of the molecule^16^,^20^,^21^,^22^,^23^,^24^,^25^, none of them have shown the complete oligomeric protein complex. The most complete structural study reported the determination of two sub-structures of *B. subtilis* Lon (*Bs*Lon), which covered part of its N-terminal domain (PDB ID:3M65) and both its ATPase and proteolytic domains (PDB ID: 3M6A)^20^ (Fig. 1a). The region linking these two domains was not determined, however, the amino acid sequence analysis suggested that it was likely to be formed by coiled-coils. This linker region seems to be crucial: an *E. coli* Lon mutated in or lacking this region exhibits severely decreased ATPase activity and disruption to both its substrate translocation and degradation abilities^26^,^27^. Recently, four X-ray crystal structures of *M. taiwanensis* LonA have been resolved, including the structure of the AP-domain bound to three ADP molecules (PDB ID: 4YPL). Interestingly, the *Bs*Lon AP-domain crystallized as an open, helically arranged hexamer when bound to six molecules of ADP, but the *Mta*Lon’s AP-domain adopted a planar conformation. Unfortunately, electron microscopy (EM) studies of Lon and its bacterial and yeast counterparts FtsH and *m*-AAA proteases have likewise failed to provide a clear picture of the overall organization of the protease^16^,^17^,^18^,^28^,^29^,^30^,^31^,^32^.

In this work, we present the first full-length structures of a LonA protease and show the importance of the N-terminal domain for its integrity. Using cryo-electron microscopy, we studied the structure of a proteolytically inactive *h*Lon S855A mutant, which retains near wild-type levels of ATPase activity. We determined the structure of this mutant after its incubation with the non-hydrolyzable ATP-analogue AMP-PNP and with ADP at resolutions of 15 Å and 21 Å, respectively. In order to study the role of *h*Lon’s N-terminal domain, we also determined the enzymatic properties of a *h*Lon protein lacking its first 270 amino acids (*h*LonΔ270) and acquired insight into its structure by a cryo-EM analysis. The deletion includes the 114 amino acids of the mitochondrial targeting pre-sequence and 156 amino acids from the N-terminal domain (Fig. 1a). Since both the enzymatic activities of *h*LonΔ270 were severely disturbed and its structure showed high variability, the missing 156 N-terminal residues are essential for the stability and proper functioning of the *h*Lon hexamer.

## Results and Discussion

### Structure of the proteolytically inactive *h*Lon incubated with AMP-PNP

The three-dimensional cryo-EM analysis of the S855A Lon at 15 Å revealed that *h*Lon, in the presence of AMP-PNP, forms an asymmetric hexamer ~230 Å long by 143 Å across. The structure can be divided into three regions, the “head”, the “neck”, and the “legs” (Fig. 2, Supplementary Figs S1 and S2a). Individual cross-sections perpendicular to the *z*-axis show signs of six-fold symmetry in the head and three-fold symmetry in the neck and legs (Fig. 2a,b), while interconnection of their centers of mass gives rise to a curve and the reconstructed structure appears slightly bent (Fig. 2c).

The barrel-like “head” has pseudo six-fold symmetry, with individual subunits arranged in a planar conformation. It could be fitted without steric clashes with six subunits from the crystal structure of *B. subtilis* Lon (PDB ID 3M6A, chain A) containing the proteolytic and the ATP-binding domains (AP-domain). The “head” therefore contains the catalytic chamber of the protein complex, which has a 20 Å diameter opening at its C-terminal side, but no opening at its N-terminal side (Fig. 2b, cross-section 3). The fitted 3M6A subunits revealed that this closure corresponds to *h*Lon’s large ATPase domain (Fig. 3) containing the aromatic-hydrophobic motif (Ar-Φ) in the axial pore loops (RTYVG), which are characteristic for ATP unfoldases^33^ and form an entry gate to the catalytic chamber in structures of compartmental proteases^33^,^34^, *T. onnurineus* LonB^35^, and in the recently acquired structure of *Mta*Lon’s AP-domain^25^. Interestingly, the *Mta*Lon 4YPL crystal structure adopted a planar conformation as well, even if it contains three ADP-bound and three nucleotide-free monomers, whereas our *h*Lon S855A was incubated with an excess of ATP-analogue AMP-PNP.

**Figure 1:**
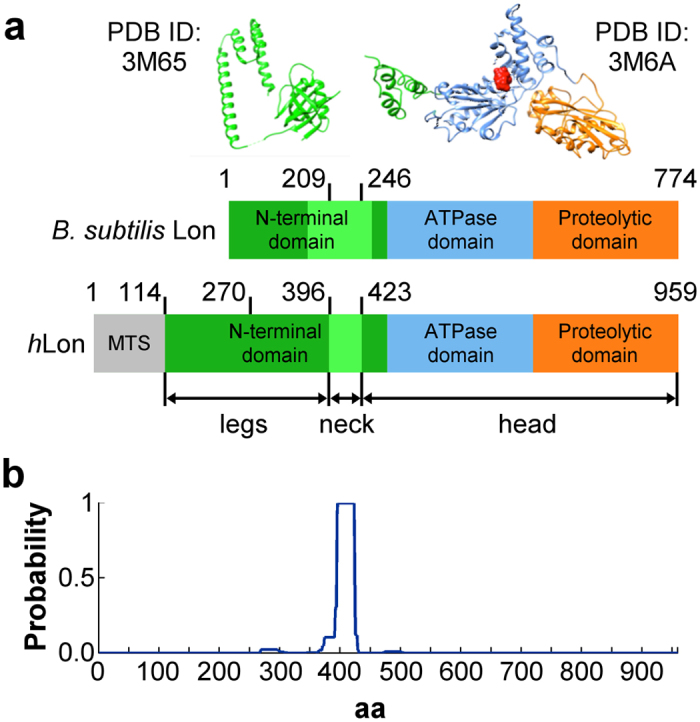
Domain arrangement of *B. subtilis* and *H. sapiens* Lon proteases. (**a**) Domain arrangement of *B. subtilis* and *H. sapiens* Lons. N-terminal domains are depicted in green, ATPase domains in blue, and proteolytic domains in orange. The mitochondrial targeting sequence (MTS) of *h*Lon is illustrated in grey, the light green regions indicate the positions of the predicted coiled-coil regions. The crystallized portions of *B. subtilis* Lon amino acid sequence (PDB IDs: 3M65, 3M6A) are illustrated as cartoons in the color code of the domain scheme, and their start and end amino acids are indicated by numbers on the top of the *B. subtilis* domain scheme. An ADP molecule crystallized with the *B. subtilis* 3M6A structure is shown in red. (**b**) COILS prediction for human Lon with a 28 amino-acid window shows that the amino acids in the region 396–423 very likely form a coiled-coil.

**Figure 2:**
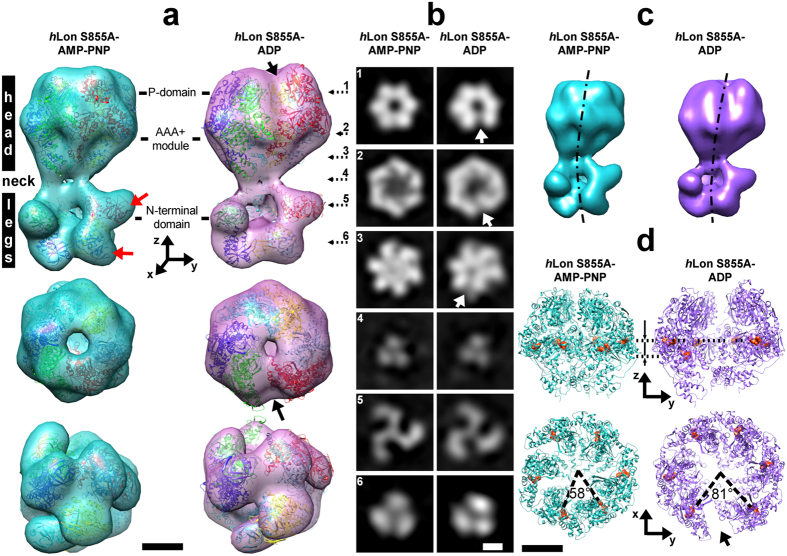
Results of cryo-EM structural analysis of a proteolytically inactive human mitochondrial Lon protease mutant (*h*Lon S855A). The structure of the AMP-PNP incubated *h*Lon S855A at 15-Å resolution is depicted in light blue, that of the ADP incubated *h*Lon S855A at 21-Å resolution is in purple. Black arrows indicate the positions of the ring opening in the ADP-bound structure, scale bars are 5 nm. (**a**) Surface representation of the reconstructed structures. Top row: side views, middle row: top views, bottom row: bottom views. Proteolytic (P) and ATPase domains (AAA^+^ module) are shown as fitted with six subunits of the *B. subtilis* Lon crystal structure (PDB ID 3M6A, chain A), the N-terminal domains are fitted with residues 1–219 of the crystal structure of the *E. coli* Lon N-terminal domain (PDB ID 3JLC). Red arrows indicate the binding sites of the sulA protein resolved in^15^. (**b**) Cross-sections through the reconstructed cryo-EM maps. Positions of the cross-section pairs within the reconstructed maps are indicated in (**a**) by dashed arrows. (**c**) Bending of the reconstructed cryo-EM maps. The approximate centers of mass of the individual cross-sections are connected by the dashed-dot lines. (**d**) Details of the *B. subtilis* Lon crystal structures as fitted into the cryo-EM maps illustrating the observed conformational changes. Top row: side views, bottom row: top-views. The ADP molecules of the crystal structure, depicted in red, indicate the planar closed ring of AMP-PNP bound Lon and the lock washer-like structure of ADP-bound Lon.

**Figure 3:**
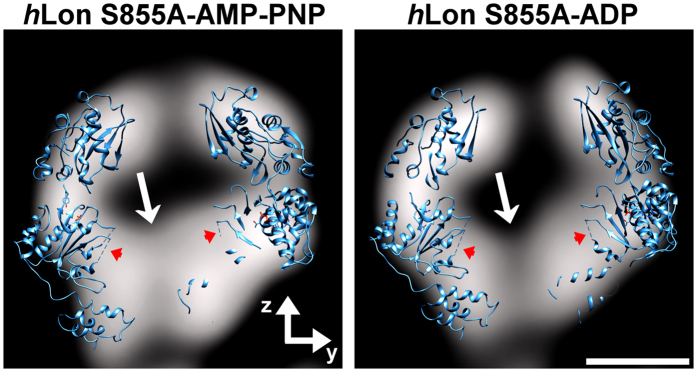
Central YZ-cross-section through the reconstructed catalytic chamber of *h*Lon S855A in binding with AMP-PNP and ADP. Orientation of the particle is identical to Fig. 2. The N-terminal tail of the catalytic chamber seems to be closed in the AMP-PNP incubated structure but it is open in the ADP-bound *h*Lon S855A structure (arrows). Two of the six fitted *B. subtilis* 3M6A monomers intersected by the plane of the cross-section, represented by blue ribbons, indicate that the density of the closure corresponds to the large ATPase domains (positions of the bridges connecting the termination points of the axial pore loops that aren’t included in the *B. subtilis* 3M6A crystal structure are indicated by red arrowheads). Scale bar: 5 nm.

The “head” narrows into a tight trimeric neck, 93 Å across and ~10 Å long, where the six Lon monomers join into three pairs (Fig. 2a,b, cross-section 4), forming a narrow, ~15 Å channel (Fig. 2b, cross-section 3, Fig. 3a). The COILS^36^ program predicted that the residues expected to occupy this region, 396–423, are likely to possess a coiled-coil arrangement (Fig. 1b). Three pairs of legs emerge from this neck in a trimer-of-dimers arrangement, giving rise to signs of a 3-fold symmetry in cross-sections through the N-terminal domains (Fig. 2b). They consist of two touching tiered-up globular densities, which could be fitted using residues 1–219 of the *E. coli* Lon N-terminal domain^21^ (PDB ID: 3LJC, Fig. 1a). This trimer-of-dimers configuration corresponds to the arrangements of the AP-domains of the ADP-bound *Mta*Lon and *T. onnurineus* LonB^25^,^35^, which arose because of the presence of low- and high-affinity ATPase sites^12^,^37^,^38^. The resolution of the reconstructed AMP-PNP incubated *h*Lon S855A structure was however not high enough to resolve the structure of the coiled-coil region and assign individual N-terminal domains to their corresponding AP-domains (Supplementary Fig. S1), indeed, the local resolution map of the reconstructed structure shows that the N-terminal domains are its most flexible part (Supplementary Fig. S1).

Interestingly, a similar pair-wise arrangement of the N-terminal helices has also been observed in the hexameric cryo-EM structure of the 26S proteasome ATPase domain^39^, and re-projections of the reconstructed *h*Lon structure (Supplementary Figs S1 and S2) give rise to the “leggy” and “leg-less” *S. cerevisiae* Lon particles^28^.

### Structure of the proteolytically inactive *h*Lon incubated with ADP

The cryo-EM map of *h*Lon S855A at 21 Å in excess of ADP showed that the barrel-shaped “head” adopted an open-ring hexameric conformation, in which six *B. subtilis* AP-domains could be fitted in a lock-washer like configuration (Fig. 2, Supplementary Figs S2b and S3). In contrast to the AMP-PNP incubated structure, the curvature of the whole ADP-incubated Lon structure is less pronounced (Fig. 2c, Supplementary Movie S6), the two monomers adjacent to the ring opening are axially displaced, and the opening angle between them increased from ~58° to ~81° (Fig. 2d). The gate to the catalytic chamber formed by the Ar-Φ loops now seems to be open (cross-section 3 in Figs 2b and 3), which suggests that ATP hydrolysis by *h*Lon induces conformational changes driving substrate translocation into the catalytic chamber, as observed in the ClpXP proteolytic machine and in the 26S proteasome^33^,^34^,^40^,^41^, and suggested also in the recent study of *Mta*Lon^25^.

Compared to the *Bs*Lon AP-domain where each of the six monomers had an ADP molecule bound^20^, the ring opening in the ADP-incubated *h*Lon structure is smaller by 13° and the axial displacement between the two monomers adjacent to the ring opening is smaller (by ~21 Å). However, the open-ring helical arrangement contrasts to the closed-ring planar arrangement of *Mta*Lon’s AP-domain with three ADPs and three nucleotide-free monomers, which may be a consequence of either a different occupancy of ADP-binding sites or restrictions imposed by crystallization. Considering the planar arrangement of the AMP-PNP incubated *h*Lon structure, it is likely that the lock-washer form of *h*Lon’s AP-domain is a true consequence of ADP binding to a full-length *h*Lon hexamer in physiological conditions. The manifold arrangements of the existing structures of Lon’s AP-domain hexamer document its flexibility, required for its allosteric operations. Lock-washer conformations were also observed in the cryo-EM structures of the D1 ring of the NSF ATPase and in the 26S proteasome^42^,^43^.

### Structure and enzymatic activities of the *h*LonΔ270 mutant

**Figure 4:**
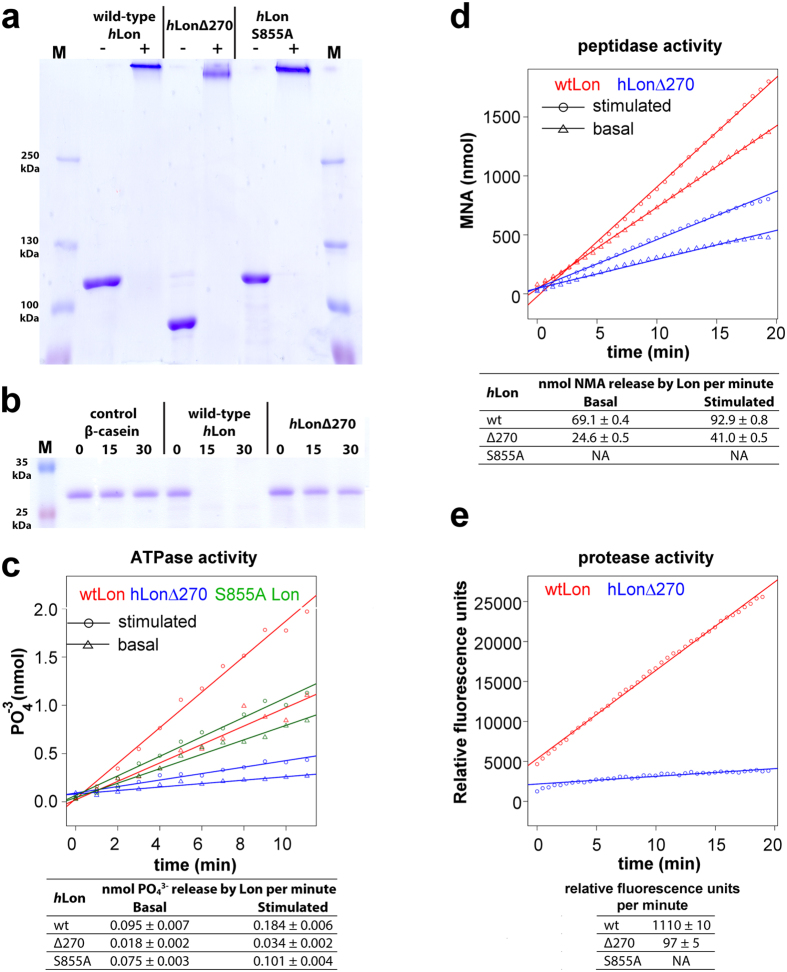
Enzymatic properties of wt *h*Lon compared to its proteolytically inactive S855A and Δ270 mutants. (**a**) Crosslinking of wild-type (wt) *h*Lon and its S855A and Δ270 mutants. 5 μg of protein was crosslinked with 0.1% (v/v) glutaraldehyde for 30 min on ice and then separated on a 5.5% SDS-PAGE gel. All Lon proteins form oligomeric structures. M: molecular marker. (**b**) Proteolytic activity of wt *h*Lon and its Δ270 mutant. 1 μg of β-casein was cleaved by 4 μg. *h*Lon for 0, 15, and 30 minutes at 37 °C. The reaction mixtures were separated on a 12% SDS-PAGE gel. The Δ270 mutant is proteolytically almost inactive. M: molecular marker. (**c**) ATPase activity of wt *h*Lon and its S855A and Δ270 mutants. The triangles (▵) and circles (⚪) show the data for the basal and β-casein-stimulated ATPase activity, respectively. The red line corresponds to the wild type, the green to the S855A mutant and blue to the Δ270 *h*Lon mutant. It can be seen that the Δ270 mutant has a much lower ATPase activity than wt *h*Lon, even lower than the S855A mutant. All three forms still show ATPase stimulation by β-casein. (**d**) Peptidase activity of wt *h*Lon and its Δ270 mutant. The triangles (▵) and circles (⚪) show the data for the basal and β-casein-stimulated peptidase activity, respectively. The red line corresponds to the wt and blue to the Δ270 *h*Lon mutant. It can be seen that the Δ270 mutant basal activity is about one-third of the wild-type, while its stimulated activity is closer to one half. The S855A mutant has no detectable peptidase activity. (**e**) Protease activity of wild type *h*Lon and its S855A and Δ270 mutants. The red line corresponds to the wt and blue to the Δ270 *h*Lon mutant protease activity. The *h*Lon Δ270 mutant protease activity is very low compared to wt *h*Lon; the S855A mutant has no detectable proteolytic activity.

**Figure 5:**
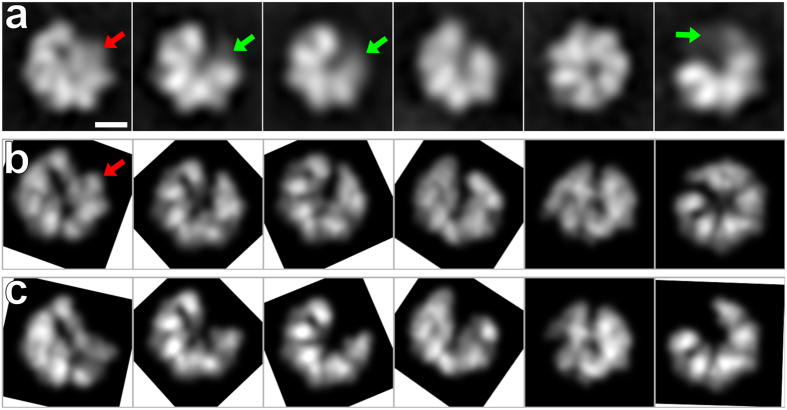
Structural heterogeneity of the shortened *h*LonΔ270 mutant. (**a**) 2D class averages of *h*LonΔ270 acquired by Relion show no sign of the N-terminal domain. Scale bar: 5 nm. Red arrows indicate features that could be uniquely assigned to a projection of a hexamer, green arrows point at decreased electron densities at the positions of the expected sixth 3M6A subunit. (**b**) Matching projections of six 3M6A subunits fitted into the AP-domain of the ADP-bound full-length Lon protein after the conversion of the x-ray structures to electron density maps. (**c**) Matching projections of five 3M6A subunits fitted into the AP-domain of the ADP-bound full-length Lon protein after the conversion of the x-ray structures to electron density maps.

In order to study the role of Lon’s N-terminal domain in its activities, we constructed a *h*Lon mutant lacking the first 270 residues, i.e. missing the 114 amino acids of the mitochondrial targeting pre-sequence and the first 156 amino acids of the mature protein. Secondary structure prediction algorithms of *h*Lon’s N-terminal domain indicated that it could be divided into two subdomains separated by an unstructured region^44^. The first subdomain, from the beginning of the protein to around residue 270 (valine), contains many β-sheets, while the region after this residue, including also the predicted coiled-coil pattern of the domain linker region (Fig. 1a), is predicted to be predominantly α-helical. We found that this shortened *h*Lon still forms a multimeric complex (Fig. 4a) and preserves a small degree of the ATPase and peptidase activities of the full-length protein, but that it has almost no proteolytic activity (Fig. 4). During the cryo-EM image analysis of this protein, we encountered a large amount of structural heterogeneity and could not detect any hint of the N-terminal domain in the acquired 2D class averages (Fig. 5a, Supplementary Figs S4 and S5). In order to interpret the class averages, we compared them to matching projections of crystal structures consisting of five and six 3M6A subunits fitted into the reconstructed ADP-bound S855A mutant (after the conversion of the crystal structures to electron densities) (Fig. 5). We observed features corresponding to the hexameric complex as well as markedly decreased intensities at the expected positions of the sixth subunit, which implies its large flexibility. Therefore, the structure of this shortened *h*Lon mutant is highly variable, which indicates that the first 156 N-terminal amino acids of *h*Lon are crucial for the stability and proper assembly of the *h*Lon hexamer.

The ATPase and peptidase activities of *h*LonΔ270 are much lower than those of the wild type, but they could still be stimulated nearly 2× by β-casein binding (Fig. 4). This suggests that β-casein is still able to bind to *h*LonΔ270 and stabilize the formed complex, which then becomes more efficient. However, *h*LonΔ270 is almost completely unable to cleave β-casein (Fig. 4), even though Mg^2+^ ions and ATP are present^16^ and it’s ATPase and proteolytic compartments preserve a significant portion of their functionality. Since the loss of functionality of *h*LonΔ270 seems to be linked to its high structural variability, the cleavage of β-casein likely requires a cooperation of all six *h*Lon subunits, which supports the recently proposed models of a coordinated substrate translocation proposed in *M. taiwanensis* Lon^25^ and the ClpX unfoldase^45^.

## Conclusions

In this work, we presented the first two structures of a Lon protease in full length, as acquired by cryo-electron microscopy, which show that ATP hydrolysis by human mitochondrial Lon protease induces conformational changes to the whole hexameric complex and stress the important role of its N-terminal domains. In particular, the N-terminal gate to its catalytic chamber appears closed by the axial pore loops when *h*Lon is incubated with AMP-PNP, but opens up at ADP binding. In addition, the *h*LonΔ270 mutant lacking the first 156 amino acids could not cleave β-casein and its 2D class averages indicated large flexibility of its sixth subunit. Therefore, proper assembly and functioning of the *h*Lon complex are guaranteed only if the first 156 amino acids of *h*Lon’s N-termini domains are present.

## Methods

### Expression and purification of recombinant proteins

Expression, purification, and *in vitro* mutagenesis of *h*Lon were performed as described in Ambro *et al*.^46^. The *h*LonΔ270 mutant (Δ1–270) was prepared by in-fusion cloning into a pOPINJ vector^47^ as an N-terminal 6× His-GST tagged protein; the following primers were used:

Lon_271_FW: AGTTCTGTTTCAGGGTACCATGGTGGAGGTAGAGAACGTTGTC

Lon_960_RV: CTGGTCTAGAAAGCTTTCACCGTTCCACGGCCAG

The construct was verified by DNA sequencing (Macrogen). Protein expression and purification was performed following the protocol for wt *h*Lon^46^. The 6 × His-GST tag was removed by overnight incubation with PreScission protease at 6 °C during protein purification according to the GE Healthcare protocol.

### Gel Filtration Analysis

Analytical gel filtration was performed with a Superose 6 10/300 GL (GE Healthcare) column using buffer A (20 mM HEPES, pH 8.0, 150 mM NaCl, 20 mM MgCl_2_, 10% (v/v) glycerol). The flow rate was 0.4 ml/min. The peak fractions were analyzed by SDS-PAGE, concentrated on Microsep Advance 100 K columns (Pall, USA) and stored at −75 °C. The concentration of protein was determined using the BCA kit (Thermo Scientific, USA).

### Crosslinking of *h*Lon

Crosslinking of *h*Lon proteins was performed as described in Ambro *et al*.^46^. Briefly, 5 μg of protein was crosslinked with 0.1% (v/v) glutaraldehyde for 30 min on ice in 50 mM HEPES, pH 8.0, 10 mM MgCl_2_, 2 mM ATP and then visualized on a 5.5% SDS-PAGE gel stained with Coomassie Brilliant Blue.

### ATPase, peptidase and protease activities of *h*Lon

ATPase, peptidase and protease assays were performed as described in Ambro *et al*.^46^. In short, to measure the ATPase activity, 5 μg of wt *h*Lon, its S855A and Δ270 mutants was incubated at 37 °C in 50 mM Tris-HCl pH 8.0, 40 mM MgCl_2_, 0.5 mM ATP and measured once a minute from 0 to 11 min. Substrate stimulation of the ATPase activity was determined by assaying the ATPase reaction in the presence of 25 μg β-casein. To estimate the peptidase activity, 5 μg of wt *h*Lon and its Δ270 mutant was incubated at 37 °C in 50 mM Tris-HCl pH 8.0, 40 mM MgCl_2_, 0.5 mM ATP with 1 mM of the fluorogenic peptide glutaryl-Ala-Ala-Phe-MNA and in the absence or presence of 25 μg β-casein. Measurements were taken every 40 s for 20 min. To measure the protease activity, 15 μg of FITC-casein were cleaved by 5 μg of wild type *h*Lon and its S855A and Δ270 mutants in 50 mM Tris-HCl pH 8.0, 40 mM MgCl_2_, 0.5 mM ATP at 37 °C. Measurements were taken every 30 s for 20 min. To measure the proteolytic activity of *h*Lon and the Δ270 mutant, 1 μg of β-casein was cleaved by 4 μg *h*Lon in 50 mM Tris-HCl pH 8.0, 10 mM MgCl_2_, 2 mM ATP for 0, 15, and 30 minutes at 37 °C. The reaction mixtures were separated on a 12% SDS-PAGE gel.

### Specimen preparation for cryo-electron microscopy

All three specimens (*h*Lon S855A incubated with 1 mM ADP or AMP-PNP, and *h*LonΔ270), concentrated to 1 mg/ml, were diluted 5× in buffer B (20 mM HEPES, pH 8.0, 150 mM NaCl, 5 mM MgCl_2_) and 3 μl of the dilution were applied to freshly glow-discharged Quantifoil 2/2 grids (EMS, Hatfield, PA, USA). The grids were vitrified by plunge-freezing into liquid ethane in a Vitrobot machine (FEI, Hillsboro, OR, USA) after 2 s blotting (blotting force 2) at 4 °C and 100% humidity.

### Data acquisition

Datasets of both *h*Lon S855A samples were acquired with a FEI Polara TEM (FEI, Hillsboro, OR, USA) at an operating voltage of 100 kV with a FEI Falcon I 4 k × 4 K direct detection camera at IGBMC Strasbourg, France. Since the overall organization of the Lon protease has been intriguing so far, we made use of the markedly better performance of the Falcon I DED at voltages lower than 200 kV in the low-resolution range of 0–~0.25 × Nyquist frequency^48^. Images were automatically recorded by the EPU software at a nominal magnification of 59000×, yielding a final image pixel size of 1.8 Å. At this magnification, the 0.25 × Nyquist frequency corresponds to the resolution of ~14.4 Å. Image underfocus was set in EPU to vary between −1.2 μm and −2.6 μm, the total electron dose used to acquire a single image was ~10 electrons/Å^2^. In total, 1074 images of ADP-incubated *h*Lon S855A and 575 images of AMP-PNP-incubated *h*Lon S855A were recorded.

The *h*LonΔ270 dataset was recorded with a C_s_-corrected FEI Titan Krios TEM at an accelerating voltage of 300 kV at IGBMC Strasbourg, France, on a Falcon II 4 K × 4 K direct-detection camera operated in movie mode. The nominal acquisition magnification was 47000×, resulting in a final image pixel size of 1.42 Å. Seven of the recorded frames (frames 2–8) covering an accumulated electron dose of ~20 electrons/Å^2^ were saved for further processing. The EPU-driven image underfocus varied between −1.3 μm and −2.4 μm; in total 2980 stacks of 7 frames were recorded.

### Image processing

After CTF and ice quality inspection, 498 images of the ADP-incubated *h*Lon S855A, 458 images of the AMP-PNP incubated *h*Lon S855A, and 2739 images of the *h*LonΔ270 specimens were selected for further processing. The *h*LonΔ270 frames were aligned and summed with the GPU-based dosefgpu_driftcorr^49^ program prior to further analysis. Image CTFs were estimated by CTFFIND3^50^, the resolution of our micrographs by the GCTF’s EPA procedure^51^, which revealed that reliable signal can be extracted from these micrographs up to a resolution, on average, of 6.74 Å for the S855A-AMP-PNP sample, 8.1 Å for the S855A-ADP sample, and 4.85 Å for the frame-aligned *h*LonΔ270 specimen. The value of the C_s_ coefficient of spherical aberration was set to 0.1 in the processing of the *h*LonΔ270 dataset. Particles were picked semi-automatically using the e2boxer program from EMAN2^52^. Altogether, 38100 *h*Lon S855A ADP-incubated particles, 97500 *h*Lon S855A AMP-PNP-incubated particles, and 39800 *h*LonΔ270 particles were extracted. The box size was set to 224 pixels for the *h*Lon S855A samples, and to 192 pixels for the *h*LonΔ270 specimen.

The collected particle sets were fully processed in Relion 1.3^53^. After three runs of 2D classification with 300 classes, 73000 *h*Lon S855A AMP-PNP-incubated particles, 32500 *h*Lon S855A ADP-incubated particles, and 12450 *h*LonΔ270 particles were selected for subsequent 3D classification. Inspection of the resulting 2D class averages of full-length specimens revealed top, side, and tilted views (Supplementary Figs S1–S3). Top views were identified according to oligomeric features^31^, while side and tilted views according to hints of the N-terminal domains.

In order to create an initial model for the 3D classification of both full-length *h*Lon S855A datasets, the identified side-view class average of the *h*Lon S855A AMP-PNP-incubated specimen was low-pass filtered to 120 Å and extended to a rotationally symmetric 3D model by rotation of its 2D Fourier transform about its long axis, followed by inverse 3D Fourier transformation. The rotation of the 2D Fourier spectrum was performed by bilinear interpolation between neighboring points of Fourier profiles at each Z-level of the Fourier-transformed side view. All these computations were performed in Matlab (Mathworks, Inc., Natick, MA, USA).

In Relion, these initial 3D models were again low-pass filtered to 120 Å and subjected to grey-scale invariant cross-correlation in the first iteration of the 3D classifications. All 3D classification runs were performed with the initial starting model, and the number of classes in each 3D classification run was selected so that at least one “empty”, i.e. very sparsely populated class, resulted.

The first 3D classification of the *h*Lon S855A AMP-PNP-incubated dataset was performed with 5 classes; 30000 particles from the most populated class were then passed through another classification run with 3 classes. From this 2^nd^ run, 23170 particles were selected for final 3D refinement. 3D classification of the *h*Lon S855A ADP-incubated dataset was performed in 3 runs with 6, 3, and 6 classes, respectively. After the 3^rd^ run, 10809 particles were available for 3D refinement. In each 3D classification run on the full-length Lon datasets, only those particles contributing to classes with well-formed N-terminal domains were selected for further 3D processing.

The 3D maps of the major classes acquired from the final 3D classification run were low-pass filtered to 40 Å and used as initial models for 3D refinement, giving an 18.3 Å resolution *h*Lon S855A–AMP-PNP structure, and 22.4 Å *h*Lon S855A–ADP structure. The reported resolutions were estimated using Relion’s gold-standard Fourier Shell Correlation at the level of 0.143. Both full-length *h*Lon S855A structures were then masked, resulting in a resolution of 15 Å for the AMP-PNP-incubated structure and 21 Å for the ADP-incubated structure. Local resolution maps were computed using ResMap^54^. The handedness of all reconstructions was adjusted to match the orientation of the *B. subtilis* Lon hexamer^20^ (PDB ID 3M6A).

In order to check the influence of the starting model on the resulting quaternary structure of the full-length protein, we processed the 23170 *h*Lon S855A AMP-PNP-incubated particles against a synthetic soft-edged cylinder with a length of 220 Å and a diameter of 140 Å. The resulting structure was identical to the one showed in Fig. 2a, albeit its resolution was slightly lower (15.5 Å).

### Fitting of X-ray structures

Rigid-body fitting of the AP-domains of the reconstructed structures was performed by the computation of cross-correlation coefficients between the reconstructed cryo-EM maps and chain A of the *B. subtilis* Lon AP-domain (PDB ID 3M6A)^20^ in the colores package^55^ of the Situs program suite^56^, followed by interactive docking in Sculptor^57^. Six AP-domain monomers could be fitted into both full-length cryo-EM S855A Lon maps without clashes (Fig. 2). The N-terminal domain of both full-length S855A Lon structures was fitted in UCSF Chimera with six copies of a fragment of the *E. coli* Lon N-terminal domain crystal structure (PDB ID: 3LJC)^21^ containing residues 1–219.

Measurements in the fitted PDB structures were performed in UCSF Chimera. In order to compare the two nucleotide-state structures of *h*Lon S855A, both reconstructed cryo-EM maps were loaded into Chimera, aligned, and fitted with the six corresponding *B. subtilis* AP-domain monomers from the Sculptor docking. The positions of the ADP molecules in the *h*Lon S855A AMP-PNP Lon reconstruction could be approximated by a virtual plane, which served as a reference also for the measurement of both vertical and angular distances between the ADP molecules in the *h*Lon S855A ADP Lon reconstruction. Distances in the cryo-EM maps were measured using ImageJ^58^. Conversion of the x-ray structures to electron density maps was performed by the e2pdb2mrc.py programme of EMAN2^52^.

## Additional Information

**Accession codes**: The reconstructed density maps of the cryo-EM *h*Lon S855A incubated with AMP-PNP and ADP have been deposited in the Electron Microscopy Data Bank under accession codes EMD-3275 and EMD-3274.

**How to cite this article**: Kereïche, S. *et al*. The N-terminal domains plays a crucial role in the structure of a full-length human mitochondrial Lon protease. *Sci. Rep.* **6**, 33631; doi: 10.1038/srep33631 (2016).

## Supplementary Material

Supplementary Movie S6

Supplementary Information
