# Supplementary material for: The N-terminal domain plays a crucial role in the structure of a full-length human mitochondrial Lon protease
Source: Sci Rep. 2016 Sep 16;6:33631. doi: 10.1038/srep33631 (PMC5025710; doi:10.1038/srep33631)
Supplement: Supplementary Information [file srep33631-s2.pdf]

The N-terminal domain plays a crucial role in the structure of a full-length human mitochondrial Lon protease

Sami Kereïche[1■], Lubomír Kováčik[1■*], Jan Bednár[1,2], Vladimír Pevala[3], Nina Kunová[3], Gabriela Ondrovičová[3], Jacob Bauer[3], Ľuboš Ambro[3], Jana Bellová[3], Eva Kutejová[3, 4*], and Ivan Raška[1]

Supplementary information

Table of contents:

1. Supplementary Figure S1

2. Supplementary Figure S2

3. Supplementary Figure S3

4. Supplementary Figure S4

5. Supplementary Figure S5

6. Supplementary Movie S6

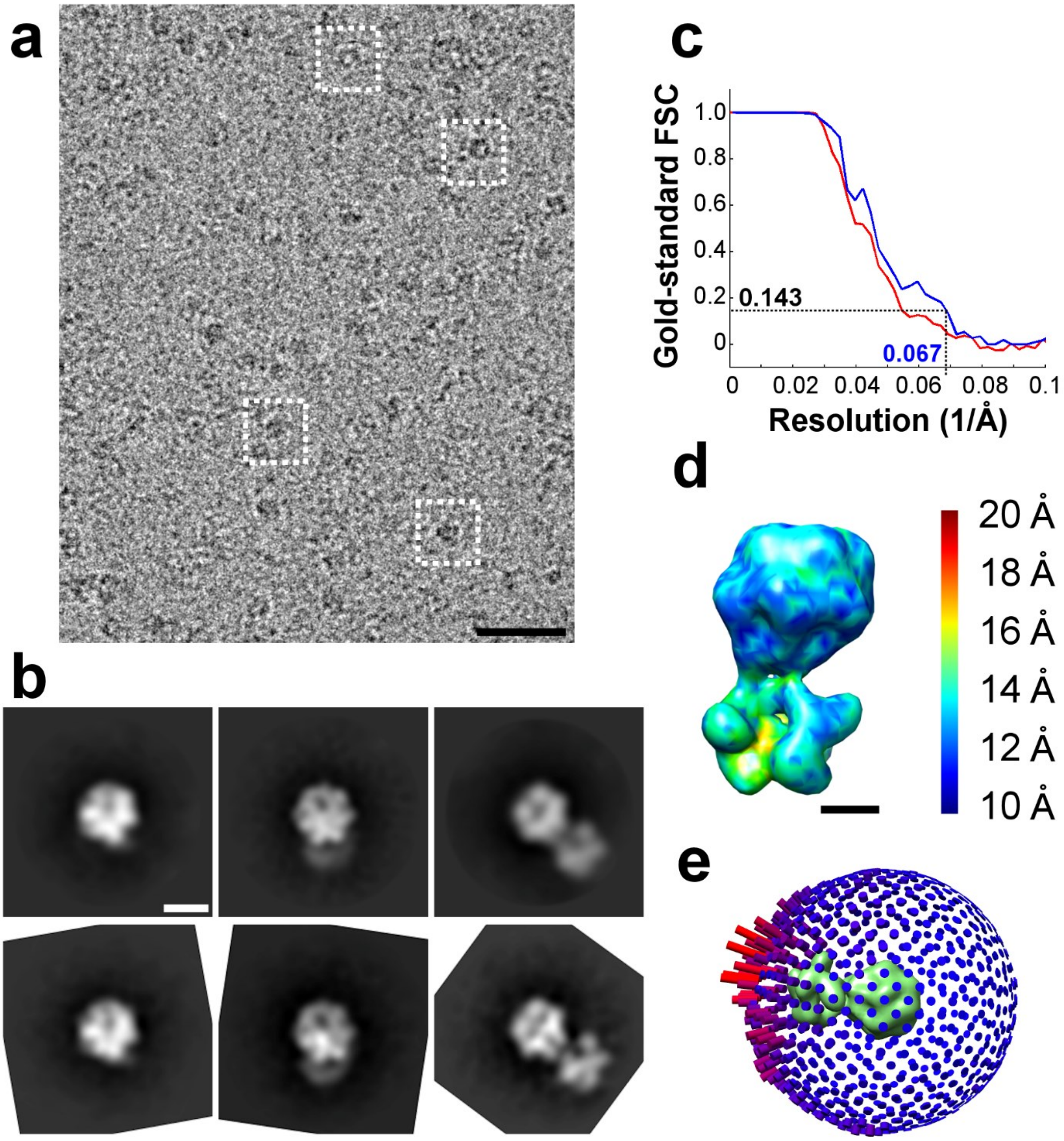

Supplementary Figure S1. Results of cryo-EM analysis of *h*Lon S855A incubated with AMP-PNP.

a) Sample micrograph captured with a FEI Falcon I detector. Typical particles are boxed. Scale bar: 50 nm. b) Top row: typical class averages resulting from Relion 2D classification. From left to right: a top view, a tilted view, and a side view. Bottom row: matching re-projections of the reconstructed 3D structure. Scale bar: 10 nm. c) Gold standard Fourier Shell Correlation curve of unmasked (red line) and masked (blue line) refined volumes, indicating a resolution of 14.9 Å at the 0.143 level. d) Local resolution map of the reconstructed cryo-EM density. Scale bar: 5 nm. e) Distribution of angular orientations of particles included in the final 3D refinement.

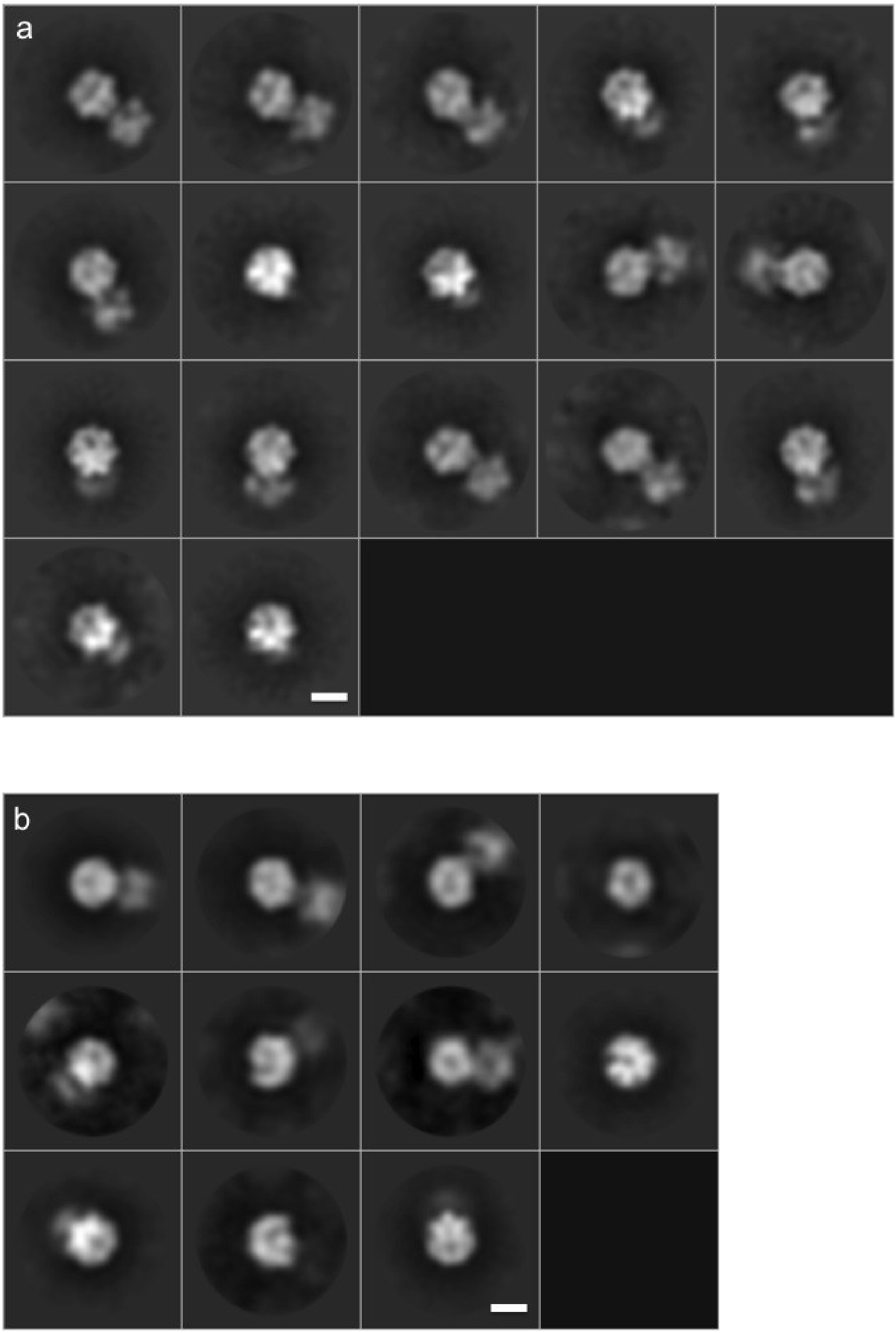

Supplementary Figure S2. Class averages after 2D classification of the *h*Lon S855A incubated with AMP-PNP (a) and ADP (b). Scale bar: 10 nm.

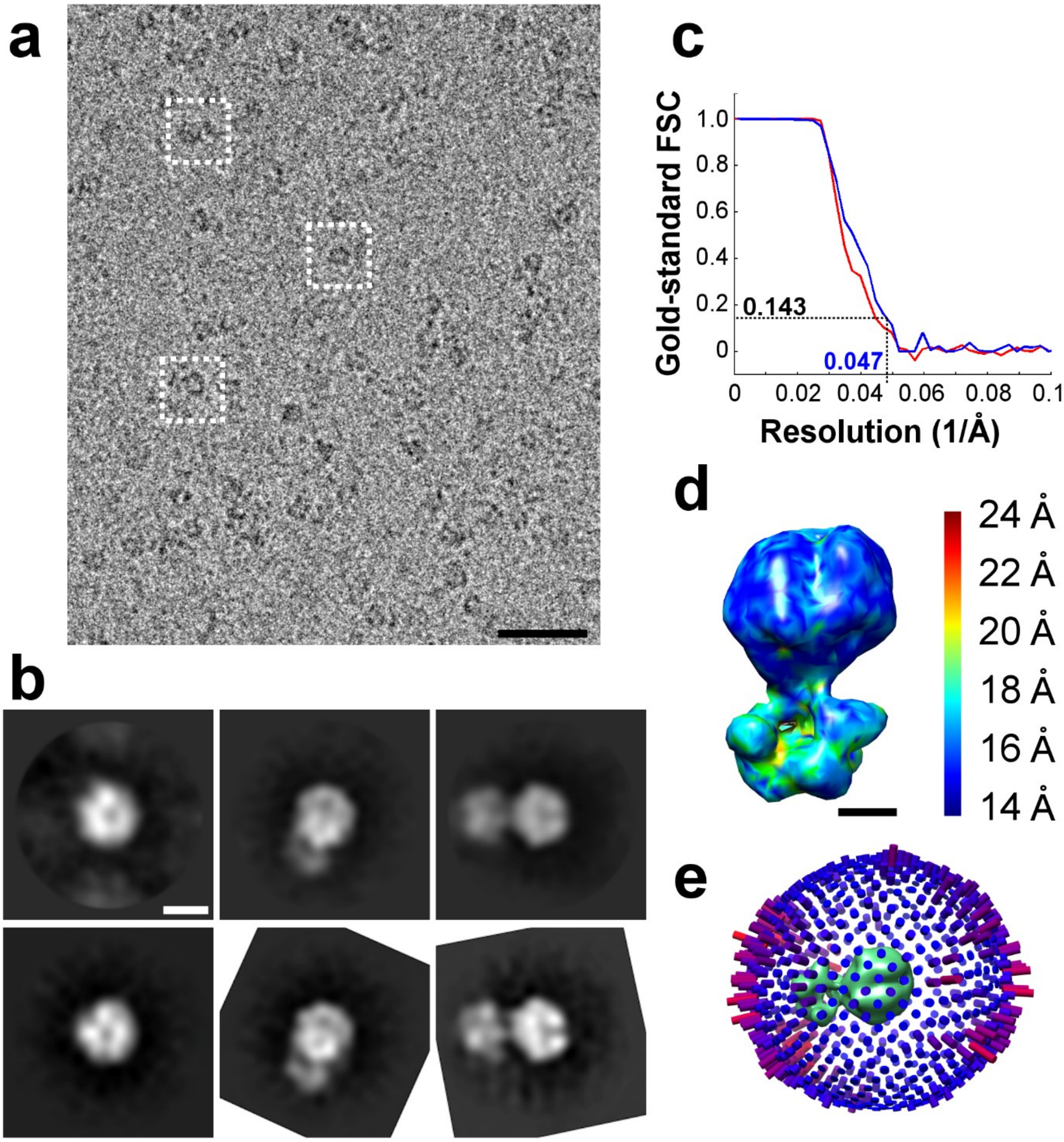

Supplementary Figure S3. Results of cryo-EM analysis of *h*Lon S855A incubated with ADP.

a) Sample micrograph captured with a FEI Falcon I detector. Typical particles are boxed. Scale bar: 50 nm. b) Top row: typical class averages resulting from Relion 2D classification. From left to right: a top view, a tilted view, and a side view. Bottom row: matching re-projections of the reconstructed 3D structure. Scale bar: 10 nm. c) Gold standard Fourier Shell Correlation curve of unmasked (red line) and masked (blue line) refined volumes, indicating a resolution of 21.3 Å at the 0.143 level. d) Local resolution map of the reconstructed cryo-EM density. Scale bar: 5 nm. e) Distribution of angular orientations of particles included in the final 3D refinement.

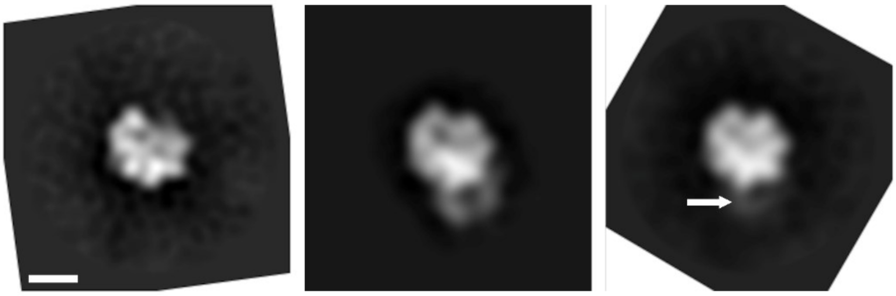

Supplementary Figure S4. The tilted-view 2D class of the shortened *h*LonΔ270 mutant lacks the mass of the N-terminal domain.

From left to right: 2D class average of the *h*LonΔ270 mutant lacking the first 156 amino acids acquired by Relion 2D classification, re-projection of the 3D structure of the full-length ADP-incubated S855A mutant in the same orientation, and a matching 2D class average of the full-length ADP-incubated S855A mutant acquired by Relion 2D classification. Scale bar: 10 nm. Arrows point at the density of the N-terminal domain present in the 2D class average of the full-length *h*Lon structure. There is no sign of such density in the corresponding projection of the shortened *h*LonΔ270 mutant. The open-ring 3D re-projection of the ADP-incubated full-length S855A mutant was calculated with EMAN2's `e2project3D.py` programme.

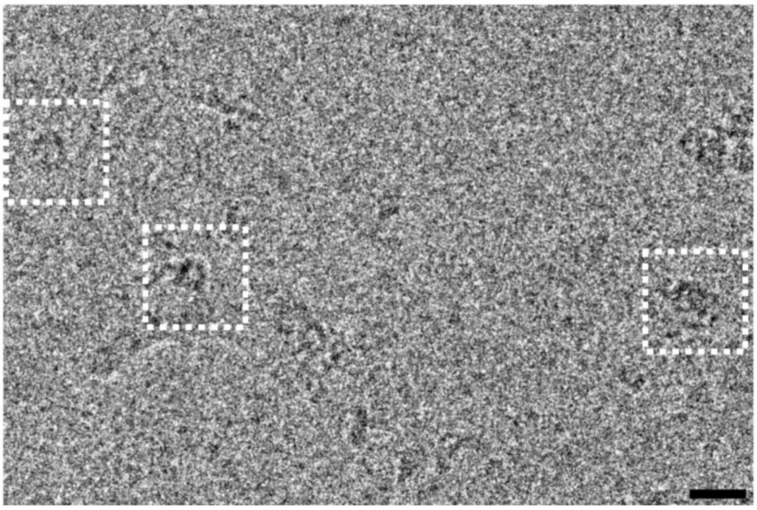

Supplementary Figure S5. Sample micrograph of *h*LonΔ270 captured with a FEI Falcon II detector. Typical particles are boxed. Scale bar: 20 nm.

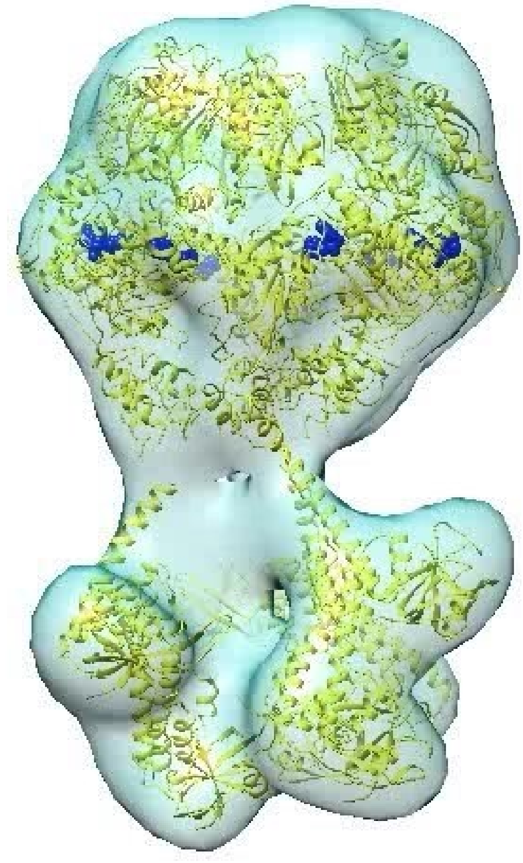

Supplementary Movie S6. Visualization of the conformational changes of the *h*Lon S855A protease induced by the binding of two different nucleotides.
